# Effect of Hypoproteic and High-Fat Diets on Hippocampal Blood-Brain Barrier Permeability and Oxidative Stress

**DOI:** 10.3389/fnut.2018.00131

**Published:** 2019-01-09

**Authors:** Cristhyane Costa de Aquino, Ricardo A. Leitão, Luís A. Oliveira Alves, Vanessa Coelho-Santos, Richard L. Guerrant, Carlos F. Ribeiro, João O. Malva, Ana P. Silva, Reinaldo B. Oriá

**Affiliations:** ^1^Laboratory of Tissue Healing, Ontogeny and Nutrition, Department of Morphology, School of Medicine, Institute of Biomedicine, Federal University of Ceara, Fortaleza, Brazil; ^2^Faculty of Medicine, Institute of Pharmacology and Experimental Therapeutics, University of Coimbra, Coimbra, Portugal; ^3^Coimbra Institute for Clinical and Biomedical Research, Faculty of Medicine, University of Coimbra, Coimbra, Portugal; ^4^CNC.IBILI, University of Coimbra, Coimbra, Portugal; ^5^Division of Infectious Diseases and International Health, Center for Global Health, School of Medicine, University of Virginia, Charlottesville, VA, United States

**Keywords:** high-fat diet, regional basic diet, blood-brain barrier, neuroinflammation, oxidative stress, malnutrition

## Abstract

Worldwide, millions of people are exposed to dietary imbalance that impacts in health and quality of life. In developing countries, like in Brazil, in poor settings, dietary habits, traditionally hypoproteic, are changing rapidly to western-type high-fat foods. These rapidly changing dietary habits are imposing new challenges to human health and there are many questions in the field that remain to be answered. Accordingly, we currently do not know if chronic consumption of hypoproteic (regional basic diet, RBD) or high-fat diets (HFD) may impact the brain physiology, contributing to blood-brain barrier (BBB) dysfunction and neuroinflammatory events. To address this issue, mice were challenged by breastfeeding from dams receiving standard, RBD or HFD from suckling day 10 until weaning. Immediately after weaning, mice continued under the same diets until post-natal day 52. Herein, we show that both RBD and HFD cause not only a peripheral but also a consistent central neuroinflammatory response, characterized by an increased production of Reactive Oxygen Species (ROS) and pro-inflammatory cytokines. Additionally, BBB hyperpermeability, accounted by an increase in hippocampal albumin content, a decrease in claudin-5 protein levels and collagen IV immunostaining, was also observed together with an upregulation of vascular cell adhesion molecule 1 (VCAM-1). Interestingly, we also identified a significant astrogliosis, manifested by upregulation of GFAP and S100β levels and an intensification of arbor complexity of these glial cells. In sum, our data show that dietary imbalance, related with hypoproteic or high-fat content, impairs BBB properties potentially favoring the transmigration of peripheral immune cells and induces both a peripheral and central neuroinflammatory status. Noteworthy, neuroinflammatory events in the hippocampus may cause neuronal malfunction leading to cognitive deficits and long-term persistence of this phenomenon may contribute to age-related neurodegenerative diseases.

## Introduction

According to the World Health Organization (WHO), about 45% of deaths, among children under 5 years old, are directly related to various degrees of malnutrition in poor settings of the middle- and low-income countries (1). Interestingly, in the same settings, overweight and obesity rates are escalating during early childhood, a scenario particularly evident in Brazilian favelas [shantytowns; (2)]. Characteristics of this dual burden are also seen largely elsewhere in low resource countries (3). Worldwide, surveillance data from WHO shows that 155 million pre-school children suffer from impairment in physical and cognitive development, while 41 million are overweight or obese (4). Obesity is particularly worrisome, since the impact in human health is expected to greatly increase in the coming years. This scenario raises public health concerns related to malnutrition, short and long-term illness and their increasing costs.

The effects of malnutrition may be endemic in low-income families. Many people cope with poor education, hygiene and precarious access to adequate health care, which could create a poor environment for optimal brain development (5). During the last decades obesity has reached epidemic proportions in developing countries, causing deaths and permanent sequelae (6). The magnitude of this burden, especially on children's cognitive development, is still mostly unknown.

Blood-brain barrier (BBB) is a dynamic and specialized structure composed of endothelial cells, connected with each other by tight (TJs) and adherens (AJs) junctions, and associated with pericytes, basement membrane and astrocyte endfeet, that together with microglia and neurons comprise the neurovascular units. The brain endothelium, with the lack of fenestrations and low fluid-phase endocytosis (pinocytosis), is the first “physical barrier” where intercellular complexes confer low paracellular permeability and high electrical resistance to the BBB (7). The core importance of the BBB is demonstrated by its role in the maintenance of brain homeostasis and protection against toxic compounds and blood composition fluctuations, but simultaneously providing essential nutrients for the normal brain function (7, 8). In fact, the presence of selective transporters allows the passage of specific molecules into the brain parenchyma that are essential for its function.

Malnutrition (hypoproteic diets) and obesity (high-fat diets) may be both associated with systemic inflammation (9, 10). Moreover, these effects may mount an environmental enteropathy vicious cycle (with microbiota and epigenetics changes) (11) that may lead to different levels of central nervous system (CNS) dysfunction, including BBB disruption (12) that is the gatekeeper of the brain. In addition, oxidized-LDL, which may be increased by malnutrition states (13), can induce apoptosis to brain endothelial cells (14). Despite these interesting observations, there is still scarcity of studies evaluating the impact of high-fat and low-protein diets (especially the latter one) on the BBB integrity and neuroinflammatory responses. Therefore, in this study, we bring novel data on mice BBB challenged by two models of malnutrition, based upon chronic feeding with a Brazilian northeast regional basic diet [RBD; (15)], and a high-fat content-diet (HFD) (16).

## Materials and Methods

### Animals

In this study, C57BL/6J pregnant female mice from Charles River, Inc. were acclimated and fed with a standard chow diet for 6 days at the Faculty of Medicine, University of Coimbra's vivarium, with free access to water and food. Thereafter, pregnant females were housed individually in nursery cages to give birth. Newborn suckling mice (standardized to 6–8 litters/dam) were randomized to three experimental groups, with challenged dams receiving either regional basic diet (RBD) or high fat diet (HFD), starting 10 days after birth delivery. The controls received the chow diet. The RBD is moderately deficient in proteins and fats (82% carbohydrates, 6% protein, 2% fat), while the HFD contains approximately 60% fat (21% carbohydrates, 18% proteins, 60% fat). On day 21, pups were weaned from their mothers and continued with the initial diet until 52th day of age. After anesthesia, mice were transcardially perfused with phosphate buffered saline (PBS) or with 4% paraformaldehyde (PFA) in PBS, and the hippocampi harvested for western blotting or total brain removed for immunofluorescence, respectively. In order to assess weight gain, mice were weighed 3 times per week. The vivarium Ethics Committee of the Faculty of Medicine of the University of Coimbra approved all the experimental protocols. The effects of the diets on BBB permeability were evaluated by assessing the presence of albumin in the brain parenchyma and the expression of hippocampal vascular unit-related claudin-5, an endothelial tight-junctional protein, collagen IV and VCAM-1 immunoreactivity. Additionally, systemic inflammation-related serum cytokines, hippocampal oxidative stress, neuroinflammation and associated astrocytic response were further investigated.

### Determination of Oxidative Stress Markers

Reactive oxygen species (ROS) levels in the hippocampus were measured by N, N-diethyl-pera-phenylenediamine (DEPPD) assay as previously described (17). In brief, 5 μL of hippocampi lysates were added to 140 μL of 0.1 M sodium acetate buffer (pH 4.8) at 37°C in a 96-well plate. Samples were taken in triplicate and 100 μL of the mixed DEPPD solution and ferrous sulfate at a ratio of 1:25 was added to each well to initiate reaction. The microtiter plate was then incubated at 37°C, for 5 min, and absorbance was measured by a spectrophotometer plate reader (Biotek, Synergy HT), at 505 nm. ROS levels were calculated from a calibration curve and expressed as hydrogen peroxide (H_2_O_2_) equivalent (1 unit = 1.0 mg H_2_O_2_/L). The calibration curve for standard solution was obtained by calculating slopes from an optical density graph.

The thiobarbituric acid reactive-species (TBARs) assay was used to assess products of lipid peroxidation, via malondialdehyde (MDA) (17). Briefly, 100 μL of tissue supernatant were incubated at Room Temperature (RT) in the dark for 1 h in a TBA solution together with butylhydroxytoluene (BHT; Sigma-Aldrich) and a catalyzer (Iron III chloride; Sigma-Aldrich). Afterwards, samples were incubated at 95–100°C for 60 min and followed by butanol extraction. The supernatants were read spectrophotometrically at 532 nm (Biotek, Synergy HT) and the concentration of MDA was calculated with respect to a calibration curve using 1,1,3,3-tetramethoxypropane (Sigma-Aldrich) (range: 0.1–83.5 μM). Results were expressed as μM/mg of hippocampal tissue.

### Enzyme-Linked Immunosorbent Assay

Blood samples were withdrawn by cardiac puncture into BD Vacutainer SST Tubes (BD Bioscience, Franklin Lakes, NJ, USA). Serum was separated by centrifugation at 1,100 × g for 15 min and stored at −80°C until analysis. The released levels of IL-1β, TNF-α, and IL-10 from the three animal groups were quantified by using an ELISA Ready-SET-Go kit (eBioscience, San Diego, CA, United States), as specified in the datasheet. The results were expressed as pg/mL.

### Western Blotting

Hippocampal tissue was processed for western blotting according to standard procedures (17, 18). Briefly, the tissue was homogenized in lysis buffer (0.15 M sodium chloride, 0.05 M Tris-base, 0.005 M ethyleneglycoltetraacetic acid, 0.5% sodium deoxycholate, 0.1% SDS and 1% X-Triton, pH 7.5) supplemented with protease inhibitor cocktail tablets (Roche Applied Sciences). Total protein content was quantified using the bicinchoninic acid method (BCA; Pierce) and stored at −20°C until further use. Afterwards, the processed samples were boiled (at 95°C) in sample buffer (Tris-HCl 0.5 M, 10.4% SDS pH 6.8) for 5 min. Different concentrations of sample, containing specific proteins, have been analyzed. Each sample was submitted to electrophoresis in polyacrylamide gel (10% and 12%), running at 130 V. Then, sample proteins were transferred to a polyvinylidene difluoride membrane (PDVF) (Millipore) for 1 h 30 min at 110 V. Following membrane blocking with BSA (5%), samples were incubated with primary antibodies (goat anti-TNF-α, 1:500, Millipore; rabbit anti-IL-1β; 1:200, Santa Cruz Biotechnology; goat anti-albumin, 1:20,000, Bethyl Laboratories; mouse anti-claudin-5, 1:100, Invitrogen; rabbit anti-caveolin-1, 1:200, Santa Cruz Biotechnology, rabbit anti-p-caveolin-1, 1:200, Santa Cruz Biotechnology; mouse anti-VCAM-1, 1:200, Santa Cruz Biotechnology; rabbit anti-GFAP, 1:2,000, Sigma-Aldrich; mouse anti-S100β, 1:500, Sigma-Aldrich), overnight, at 4°C, followed by incubation with alkaline phosphatase-conjugated secondary antibody anti-goat IgG (1:10,000, GE Healthcare), anti-rabbit (1:20,000, GE Healthcare), or anti-mouse (1:10,000, GE Healthcare), 1h at RT. Internal control for protein content in each sample was evaluated by mouse anti-GAPDH (1:10,000, Sigma-Aldrich). Targeted protein bands were visualized following membrane incubation with enhanced chemifluorescence (ECF, Amersham) using a Typhoon FLA 9000 (GE-Healthcare Biosciences) and quantified using Image Studio software (version 5.2, Licor).

### Immunofluorescence

Following perfusion with PFA, brains were removed and incubated overnight with 4% PFA, followed by 30% sucrose (in PBS 0.01 M) for 24 h, at 4°C, and then stored at −80°C, until further use. Brains were cryosectioned at 20 μm thick coronal slices and mounted on glass immunoslides, washed with PBS solution, then permeabilized with Triton X-100 (1%), and blocked with 3% bovine serum albumin for 1 h at RT. Primary antibodies incubation was performed during 24 h, at 4°C, using a monoclonal mouse anti-GFAP-Cy3 conjugated antibody (1:500, Sigma-Aldrich), and rabbit anti-collagen IV (1:200, Abcam). After that, brain slices were incubated with 5 μg/mL Hoechst 33342 (Sigma-Aldrich) for 5 min in the dark at RT, for nuclei staining. Sample preparations were mounted in Dako fluorescence medium (Dako, Glostrup, Denmark) and images acquired and processed using a Carl Zeiss LSM 710 Meta confocal microscope (Carl Zeiss; Oberkochen, Germany). Quantification of GFAP and collagen IV immunoreactivity was accomplished using the NIH ImageJ 1.47 analysis software. In brief, all photograph area was considered as well as three different zones without staining (black) to be used for background subtraction. To determine the corrected total fluorescence, we used the following formula: correct total fluorescence = (integrated intensity) – (area of picture × mean background). The results are expressed as the mean of fluorescence intensity (arbitrary units) of five brain slices obtained from three different animals for each experimental group.

### Morphological Analysis of Astrocytic Processes

GFAP-labeled astrocytes were analyzed as previously described (19). Briefly, images were uploaded to the Simple Neurite Tracer plugin of FIJI Software to measure the length (μm) and count the number of astrocytic processes. Further, to infer about the arbor complexity of astrocytes a Sholl analysis was performed. A total of 30 cells were analyzed (10 cells/animal from a total of three different animals for each experimental group).

### Statistics

Data represent the mean value ± standard error of the mean (SEM) a minimum of 6 animals per group. Statistical analysis of weight gain and Sholl analysis was performed using two-way ANOVA followed by Dunnett's multiple comparisons test. Regarding all the other experimental approaches, the statistical analysis was performed using Kruskal-Wallis test, followed by Dunn's *post-test* for multiple comparisons. All statistics were calculated using GraphPad Prism 6.0. The level of significance was *P* < 0.05.

## Results

**Figure 1:**
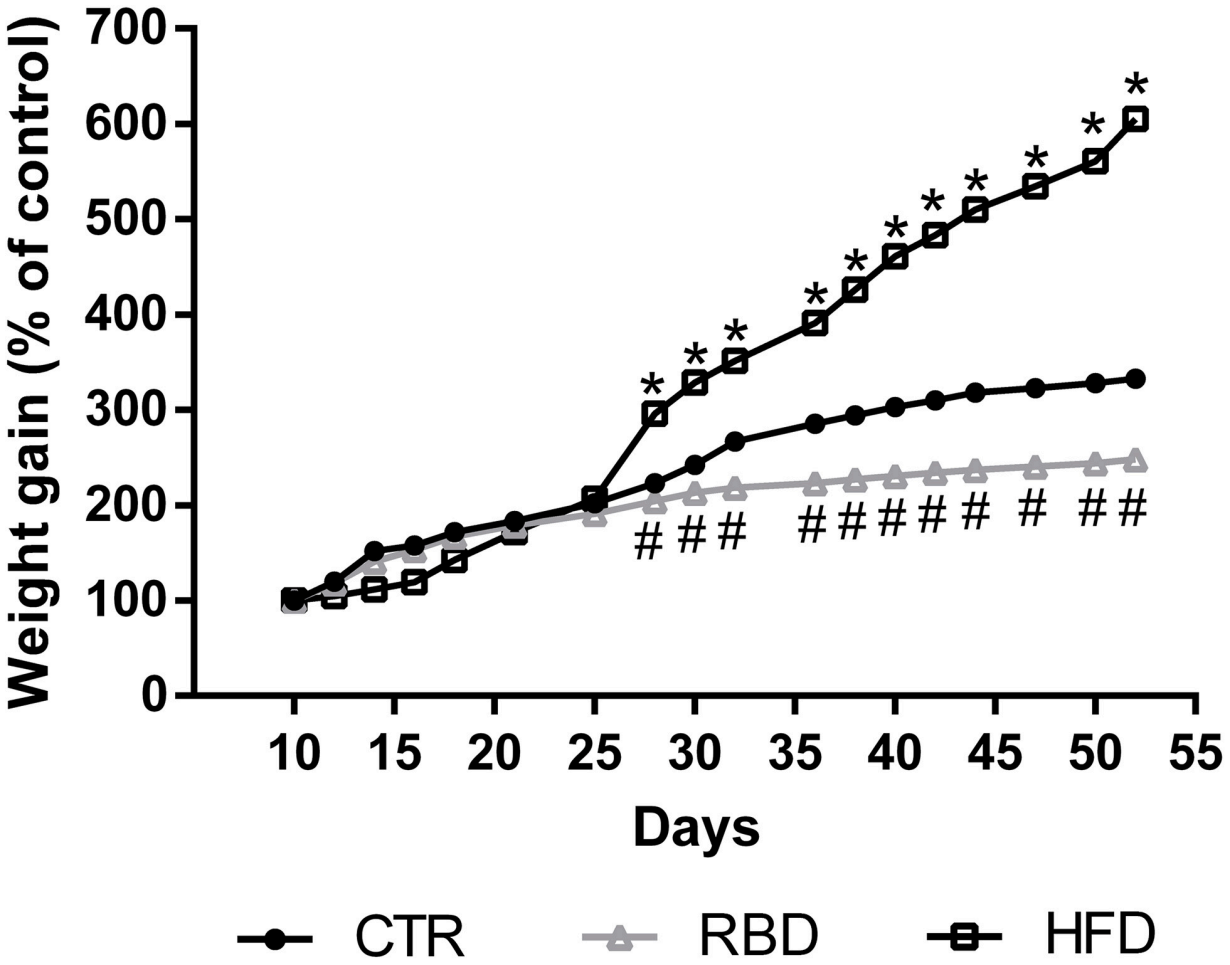
Effect of regional basic (RBD) and high-fat (HFD) diets feeding on mice body weight from postnatal day 10 to postnatal day 52. Data were analyzed by using two-way ANOVA followed by Dunnett's multiple comparisons test. ^*^P < 0.0001 and ^#^*P* < 0.0001 vs. nourished control group (CTR). The results are shown as mean ± SEM.

Mice were grouped in three cohorts submitted to control diet (*n* = 7), RBD (*n* = 8) and HFD (*n* = 8). Body weight was monitored every 2 days for a total of 42 days, starting at postnatal day 10 (mean body weight = 4.4 ± 0.3 g), until day 52 of age. Results showed that until post-natal day (PND) 25, all groups present a similar development. After that, we observed that control group developed, as expected, with a slow and sustained weight gain, reaching a mean value of 14.9 ± 0.2 g (332.7 ± 4.9% of control). Regarding RBD group, the body weight slightly increased over time reaching 10.6 ± 0.05 g (248.5 ± 1.2% of control) at 52 days of age, whereas there was a marked increase in body weight gain for the HF diet group reaching 26.2 ± 0.2 g (605 ± 6.1% of control; Figure 1).

**Figure 2:**
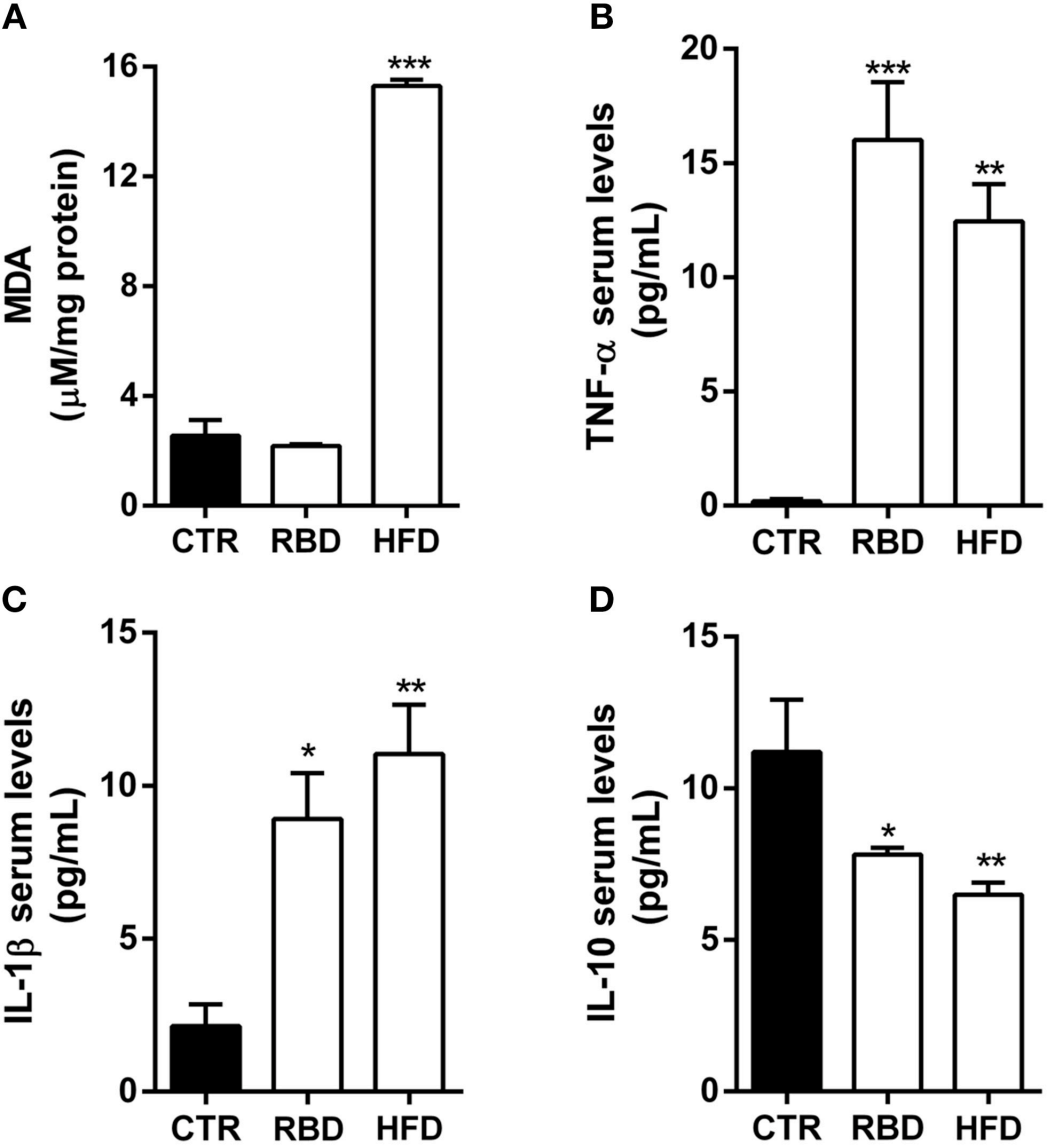
Effect of regional basic (RBD) and high-fat (HFD) diets on inflammatory mediators. **(A)** Malondialdehyde (MDA) formation, a marker of oxidative stress in hippocampal tissue, is only increased in HFD animals. ELISA quantification on blood serum protein levels of pro-inflammatory cytokines **(B)** TNF-α and **(C)** IL-1β shows an upregulation after chronic exposure to both diets. Additionally, ELISA quantification of the anti-inflammatory cytokine **(D)** IL-10 shows a significant decrease of its protein levels in both diet feeding animals. Data were analyzed by using Kruskal-Wallis test, followed by Dunn's *post-test* for multiple comparisons, *n* = 5–8. *P < 0.05, **P < 0.01, and ***P < 0.001 vs. nourished control group (CTR). The results are shown as mean ± SEM.

Afterwards, we evaluated several inflammatory mediators in blood serum of these animals. First, we observed a significant increase in lipid peroxidation products (measured by MDA reaction products) in HFD animals (Figure 2A, 15.3 ± 0.2 μM/mg; *P* < 0.001), with no effect on RBD diet animals (Figure 2A; 2.18 ± 0.1 μM/mg). Regarding pro-inflammatory cytokines, there was an increase in both TNF-α and IL-1β serum levels not only in RBD (Figures 2B,C; 16 ± 2.5 pg/mL TNF-α*, P* < 0.001; 8.9 ± 1.5 pg/mL IL-1β, *P* < 0.05; respectively), but also in HFD animals (Figures 2B,C; 12.5 ± 1.6 pg/mL TNF-α *P* < 0.01; 11 ± 1.6 pg/mL IL-1 β, *P* < 0.01, respectively). Moreover, the serum levels of the anti-inflammatory cytokine IL-10 decreased with both diets (Figure 2D; RBD 7.8 ± 0.2 pg/mL IL-10, *P* < 0.05; HFD 6.5 ± 0.4 pg/mL IL-10, *P* < 0.01). Such results clearly show that both diets induce a peripheral pro-inflammatory profile.

**Figure 3:**
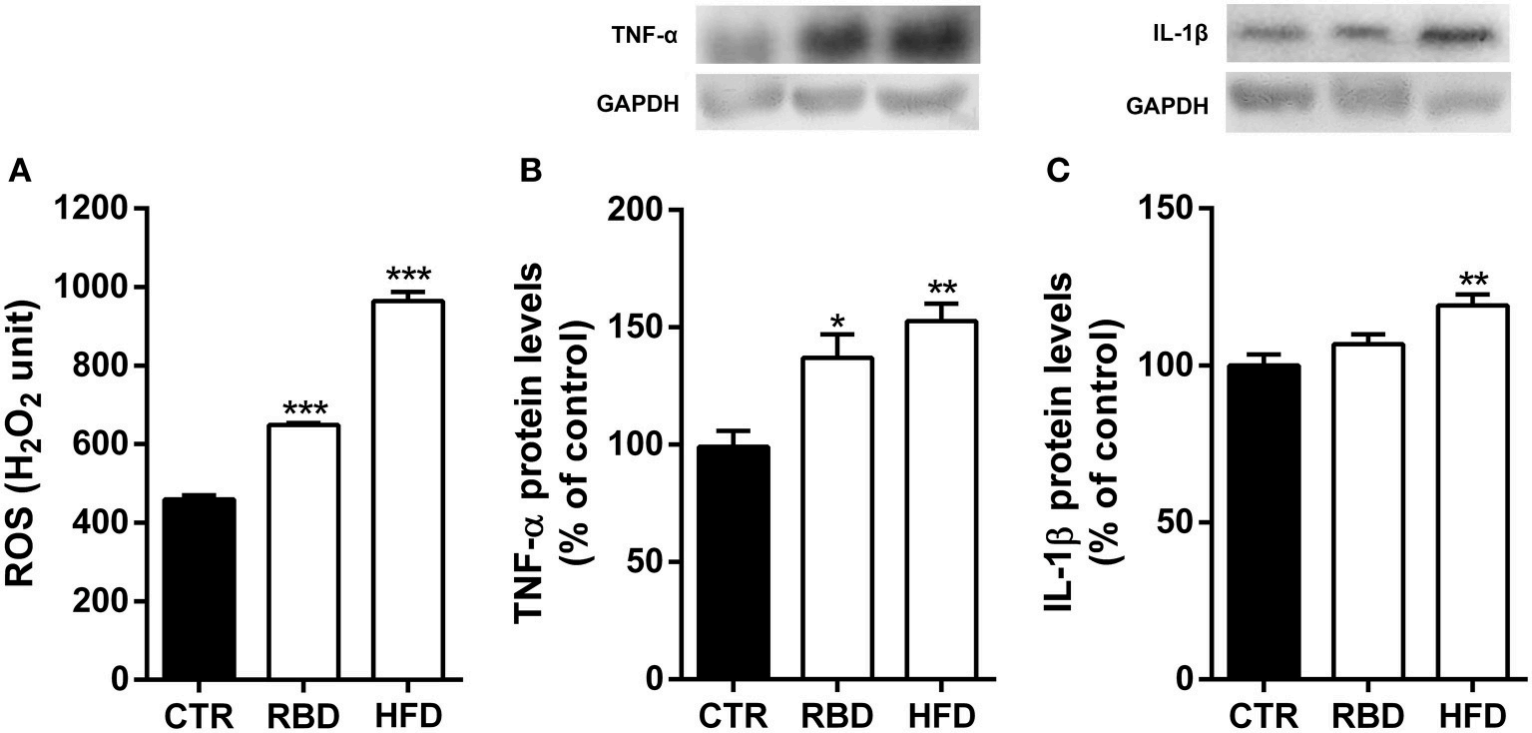
Effect of regional basic (RBD) and high-fat (HFD) diets on hippocampal neuroinflammatory response. Both diets induced an **(A)** oxidative stress observed by an increase in reactive oxygen species (ROS) and **(B)** an increase of TNF-α protein levels in mice hippocampal homogenates. **(C)** Only HFD feeding animals show a significant upregulation of IL-1β protein levels. Above the bars, representative western blot images of TNF-α (19 kDa), IL-1β (17 kDa) and GAPDH (37 kDa) are shown. Data were analyzed by using Kruskal-Wallis test, followed by Dunn's *post-test* for multiple comparisons, *n* = 5–7. *P < 0.05, **P < 0.01, and ***P < 0.001 vs. nourished control group (CTR). The results are shown as mean ± SEM.

It has already been demonstrated that blood composition fluctuations can have an impact on brain function. Therefore, we also evaluated the inflammatory status of hippocampi of the animals feed with both diets. In fact, we observed a significant increase in ROS production (Figure 3A; RBD 648.5 ± 4.7, *P* < 0.001; HFD 964.3 ± 23.3, *P* < 0.001) and TNF-α protein levels (Figure 3B; RBD 137 ± 10% of control, *P* < 0.05, HFD 153 ± 7.6% of control, *P* < 0.01) by both diets. Regarding IL-1β of its protein levels with HFD and only a tendency, yet not significant, by RBD diet (Figure 3C, 119 ± 3.5% of control, *P* < 0.01). Overall, we concluded that both diets triggered a peripheral inflammatory state that was accompanied by a concomitant hippocampal neuroinflammation.

**Figure 4:**
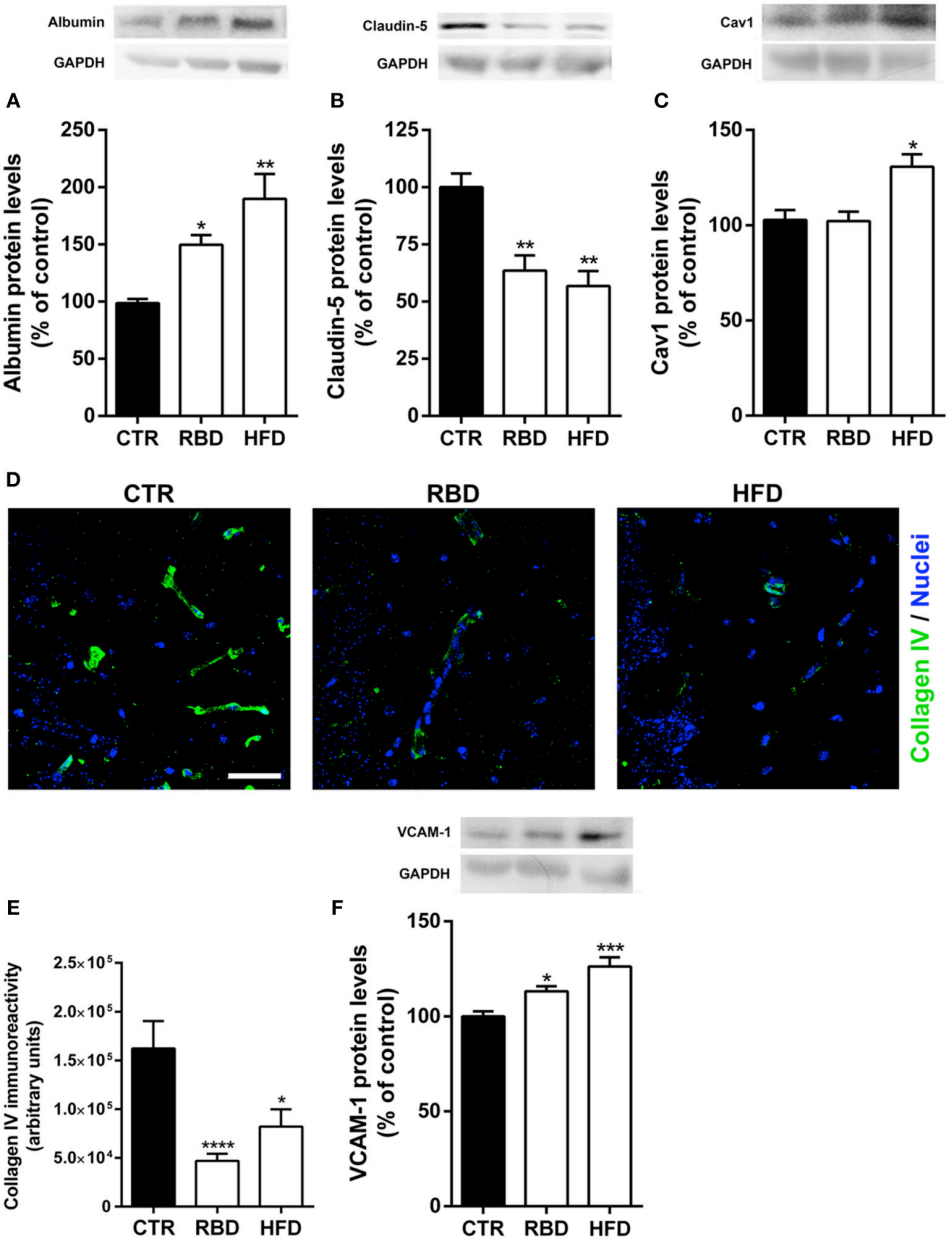
Effect of regional basic (RBD) and high-fat (HFD) diets on blood-brain barrier properties. An increase of albumin **(A)** and a decrease of claudin-5 **(B)** protein levels were observed with both RBD and HFD diets. **(C)** Regarding caveolin-1 (Cav1) protein levels, there was also a significant increase of its total levels in HFD group. **(D)** Representative immunohistochemistry images for Collagen IV (green, brain vessels), and Hoechst 33342 (blue, nuclei). Scale bar = 40 μm. **(E)** DRB and HFD induced a significant decrease in collagen IV immunoreactivity. Moreover, both diets induced a significant upregulation in **(F)** VCAM-1 (vascular cell adhesion molecule-1). Above the bars, representative western blot images of Albumin (66 kDa), claudin-5 (25 kDa), caveolin-1 (20 kDa), VCAM-1 (110 kDa), and GAPDH (37 kDa) are shown. Data were analyzed by using Kruskal-Wallis test, followed by Dunn's *post-test* for multiple comparisons, *n* = 5–7. *P < 0.05, **P < 0.01, ***P < 0.001, ****P < 0.0001 vs. nourished control group (CTR). The results are shown as mean ± SEM.

The (neuro)inflammatory processes have been described as key players in BBB disruption and transmigration of peripheral immune cells into brain parenchyma (7, 20). To evaluate BBB alterations induced by RBD and HFD, we measured the protein levels of albumin in brain tissue, since this blood-borne protein does not cross the BBB under normal conditions. Interestingly, albumin content in the hippocampus was significantly increased in mice chronically exposed to RBD (Figure 4A; 139.3 ± 10.5%; *P* < 0.05) and HFD (Figure 4A; 179.4 ± 25.3%; *P* < 0.01), indicating a hyperpermeability of BBB triggered by both diets. To further unravel the transport across the BBB that is being altered, we analyzed the protein levels of endothelial claudin-5 and caveolin-1 (Cav-1), the latter has been associated with brain injury-related BBB breakdown (21). The transport at the BBB can occur through paracellular and transcellular pathways, with the first being controlled by intercellular complexes present between adjacent endothelial cells, such claudin-5 a tight junction (TJ) protein, and the second pathway occurs through the endothelial cell and involves the formation of vesicles, including caveolin-1-coated vesicles. Herein, we observed a significant downregulation of claudin-5 protein levels (Figure 4B) in both RBD (63.5 ± 6.7%, *P* < 0.01) and HFD animals (56.8 ± 6.5%, *P* < 0.01), suggesting an increase in paracellular permeability of the BBB. After that, we investigated the effect of diets on Cav-1 and concluded that only HFD was able to induce a significant upregulation of its protein levels (Figure 4C, 131 ± 6.5%, *P* < 0.05). Further, to corroborate the weakening of BBB we also evaluated alterations in collagen IV (coll IV) immunoreactivity. This protein is one of the most abundant in basal membrane, that gives support to brain microvessels. In fact, there was a clear reduction in the staining induced by both diets (Figures 4D,E; CTR 162,180 ± 28,098; RBD 46,959 ± 7,285, *P* < 0.001; HFD 82,098 ± 17,780, *P* < 0.05).

Since BBB has also a key role in restrain the transmigration of peripheral immune cells into brain parenchyma, we further analyzed the protein levels of vascular cell adhesion protein 1 (VCAM-1), an adhesion molecule that promotes the passage of peripheral immune cells across BBB. Interestingly, chronic exposure to both diets induced an upregulation of VCAM-1 (Figure 4F; RBD 113 ± 2.7%, *P* < 0.05; HFD 126 ± 4.9%, *P* < 0.001).

**Figure 5:**
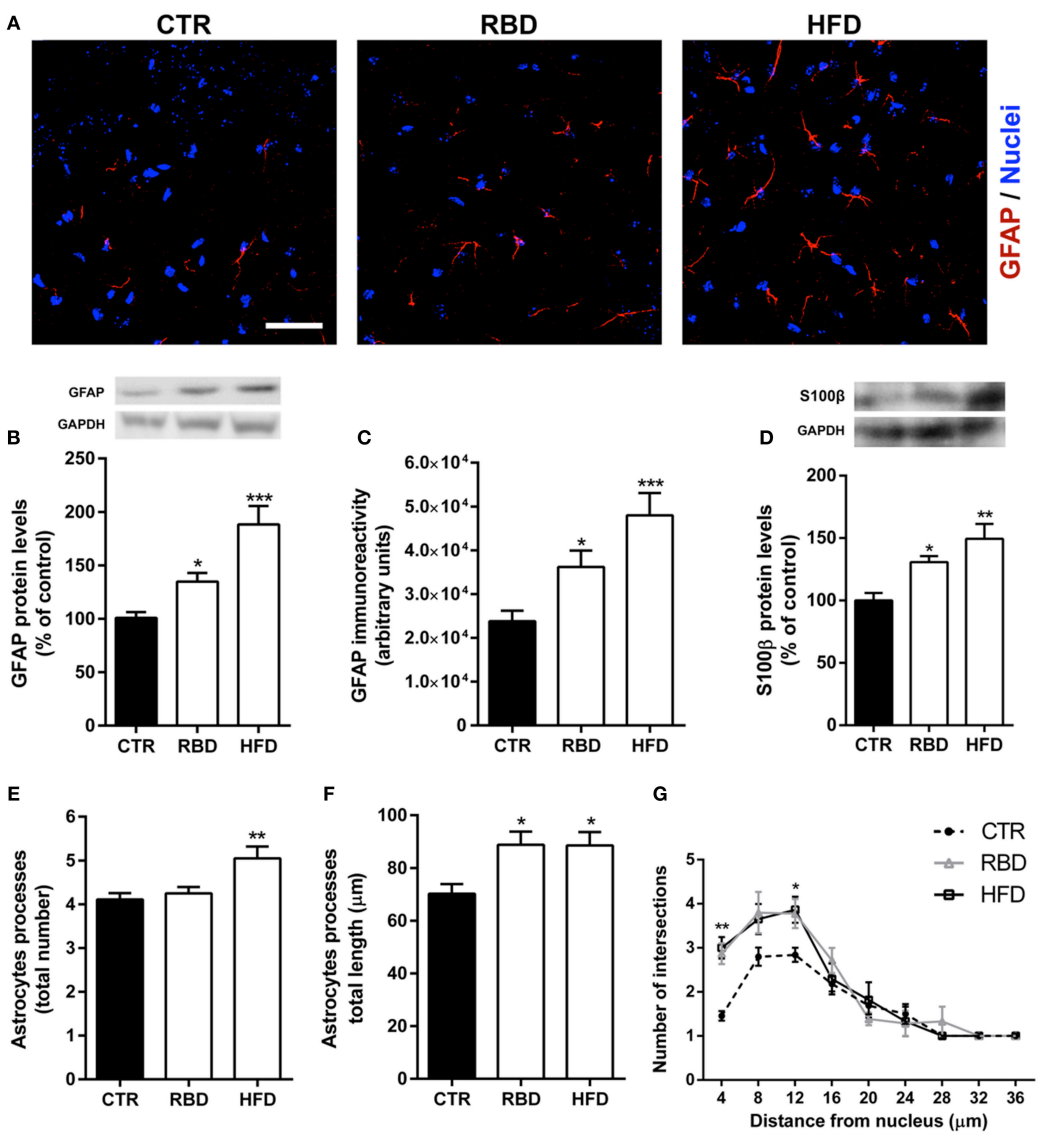
Effect of regional basic (RBD) and high-fat (HFD) diets on astrocytes properties. Representative immunohistochemistry images for **(A)** GFAP (red, astrocytes), and Hoechst 33342 (blue, nuclei). Scale bar = 40 μm. Both diets induced an increase in **(B)** GFAP protein levels, **(C)** immunoreactivity, and **(D)** S100β protein levels. Yet, HFD had a more pronounced effect than RBD. Regarding morphological alterations, only HFD increased the **(E)** total number of processes, with no effect on RBD animals. However, both diets induced a significant increase in **(F)** the total length of such processes and **(G)** on arbor complexity. Results are expressed as mean + S.E.M., *n* = 5–7 [for **(B,D)**], *n* = 10 [for **(C,G)**], *n* = 30 [for **(E,F)**]. Data were analyzed by using Kruskal-Wallis test, followed by Dunn's *post-test* for multiple comparisons. *P < 0.05, **P < 0.01, and ***P < 0.001 vs. nourished control group (CTR). A two-way ANOVA followed by Bonferroni's Multiple comparison test was used for Sholl analysis, where *P < 0.05 and **P < 0.01 vs. CTR.

Data presented herein shows that malnutrition related with RBD or HFD triggers hallmarks of oxidative stress, (neuro) inflammation, and BBB permeability. Importantly, astrocytes have a crucial role in BBB structure and function (22), as well as in neuroinflammatory processes (23). Thus, we investigated the effect of both diets on astrocytes and concluded that HFD induced a significant increase in GFAP protein levels (Figures 5A,B; 188 ± 17.3% of control, *P* < 0.001) and immunoreactivity (Figures 5A,C; 47,997 ± 5,101, *P* < 0.001, when compared with the control 23,784 ± 2,412) in the hippocampus. RBD had also an effect on both parameters analyzed, but not so significant when compared to HFD (Figure 5B, 135 ± 8.2% of control, *P* < 0.05; Figure 5C, 36,195 ± 3,758, *P* < 0.05, when compared with the control 23,784 ± 2,412). Moreover, when the protein levels of S100β, a specific marker of astrocytes reactivity, were measured, an upregulation was observed after exposure to both diets (Figure 5D; RBD 131 ± 4.79% of control, *P* < 0.05, HFD 149 ± 11.9% of control, *P* < 0.01). Taking into consideration the importance of a simultaneously analysis of cellular morphological alterations, we further concluded that both diets induced an increase in the total length of astrocytic processes (Figure 5F; CTR 70.2 ± 3.6 μm; RBD 88.8 ± 5 μm, *P* < 0.05; HFD 88.6 ± 5; *P* < 0.05), and promoted an arbor complexity of these cells (Figure 5G; *P* < 0.05). Curiously, only in HFD animals was possible to observe an increase of the total number of astrocytic processes (Figure 5E; CTR 4.1 ± 0.1; RBD 4.3 ± 0.1; HFD 5.1 ± 0.3; *P* < 0.01).

## Discussion

In recent years, a westernization of diets, with an increase in fat content, on developing countries has been observed, and has a huge socio-economic impact (24). Despite several preclinical studies exploring this issue, there is a scarcity of information regarding the impact of protein malnutrition on BBB properties. The BBB is known as the gatekeeper of the brain, being extremely selective and so protecting the brain from variations in blood composition and toxins. Nevertheless, it has also a role in providing essential nutrients for proper brain function. Thus, BBB dysfunction may lead to brain damage and indeed is known to be a common feature in all neurodegenerative diseases. Moreover, under aggression related with trauma, ischemia, metabolic disorders or malnutrition, neuroinflammatory cascades are initiated involving glial cells and molecular pathways that may interfere with BBB function (12, 17, 18, 25). In fact, a chronic long-term inflammatory environment in the brain usually leads to chronic impairment of the BBB, which will contribute to long-term neurodegeneration and neuropathology, including neurodegenerative conditions like Alzheimer's disease (26). In addition, long-term undernutrition (27), similar to our RBD and HFD feeding protocol (28, 29), induces a systemic inflammation with brain deleterious effects, being the hippocampus one of the most susceptible brain regions (29). Curiously, calorie intake, meal frequency, texture and content seem to be associated with modification in hallmarks of hippocampal physiology and cognitive performance associated with neurogenesis (30). It was recently shown that a preexisting protein malnutrition worsens the motor deficits observed after stroke (31). Moreover, post-ischemia protein malnutrition can aggravate neuroinflammatory processes and inhibits neuroplasticity in the hippocampus (32). Accordingly, in our chronic model of malnutrition or high-fat content diet we also observed both peripheral and central inflammatory markers, with an increase in both ROS production and pro-inflammatory cytokines.

Long-term feeding with RBD (27, 33) and HFD (34) may lead to intestinal barrier leakage with increased gut-to-blood bacterial translocation, and continued LPS systemic circulation. Alterations in the gut microbiota toward more LPS-releasing Gram-negative bacteria by both diets may contribute to low-grade systemic inflammation with brain deleterious effects (27, 28), including BBB disruption and oxidative stress (35). In addition, systemic inflammation could lead to BBB dysfunction by increased myeloperixodase (MPO)-releasing neutrophil activity (36). This potential MPO release from circulating blood neutrophils may be due to increased LPS binding to the endothelial surface of the brain capillaries (37). Interestingly, rats challenged with cecal ligation and puncture (as a model of sepsis) showed increased BBB permeability to Evans Blue brain staining and reduced endothelial claudin-3 and claudin-5 levels, effects that were ameliorated by the cholesterol-lowering drug simvastatin (38).

It is also known that systemic inflammation can lead to BBB dysfunction by increasing the activity of proinflammatory cytokine-releasing neutrophils (36), white blood cells that are an essential part of the innate immune system. In fact, it has been reported that HFD contributes to BBB disruption either in young and aged mice, as shown by increased permeability of brain microvessels to IgG and to sodium fluorescein and changes in tight junction proteins including occludin and claudin-5 (39, 40). Further, the same authors concluded that HFD triggers neuroinflammation, as evaluated by reactive microglia and release of pro-inflammatory cytokines (40). Moreover, in an animal model of HFD for 9 weeks, an increase in leukocyte transmigration into the brain parenchyma was observed (41). Accordingly, in the present study we showed a downregulation of collagen IV, the most important protein of the BBB basement membrane, and the tight junction protein, claudin-5, which is strong indicator of BBB dysfunction. Moreover, there was an increase in VCAM-1 protein levels, an adhesion molecule specifically expressed in endothelial cells and necessary to immune cells transmigration into the brain parenchyma. Therefore, hippocampal Cav1 immunoreactivity was increased, suggesting active endothelial transcytosis, further supporting the BBB hyperpermeability induced by the diets. Thus, we showed that both RBD and HFD feeding protocols induced a very robust (neuro) inflammatory process as well as a BBB hyperpermeability.

Glial cells, such as astrocytes and microglia, are involved in neuroinflammatory responses playing also an important role in BBB function. Tucsek et al. (40) concluded that HFD triggers neuroinflammation, demonstrated by activated microglia and release of pro-inflammatory cytokines (40). In addition, mice chronically feeding with a HFD has been associated with increased oxidative stress and GFAP in the cerebral cortex and in the hippocampal dentate gyrus with impaired recognition memory (42). These data support our observation that particularly HFD chronic consumption causes a significant astrocyte reactivity in the hippocampus, as shown by increased expression of GFAP and S100β. Nevertheless, RBD had also an effect but not so strong as with HFD. Curiously, Feoli et al. (43) did not detect alterations in hippocampal GFAP and S100β immune contents on the 60th postnatal day in undernourished rats. Such contradiction can be explained by differences related with diet feeding protocols and animal strains. Still, the authors observed an increase of S100β levels in the cerebrospinal fluid (43).

The evaluation of astrocytic morphological alterations is of crucial importance to understand the role of these glial cells on the brain function. Thus, in order to stratify the possible different types of astrocytes, a classification of A1 and A2 astrocytes has been arisen by Ben Barres lab based on different gene expression profiles (44–46). Both A1 and A2 astrocytes are reactive glial cells, in which A1 type seems to be induced by exposure to IL-1α, TNF-α and complement proteins (45). Therefore, we can hypothesize that our malnutrition models are shifting astrocytes to an A1 profile. Accordingly, we observed a significant astrogliosis proved by an increase of GFAP and S100β expression, and by significant morphological alterations of astrocytes. Still, a genomic analysis is necessary to correctly classified the type of reactive astrocytes.

In summary, the present report shows that RBD and HFD chronic feeding impacts BBB permeability, and leads to brain oxidative stress and neuroinflammation with a prominent role of astrocytes. Systemic inflammation was also observed and may underly central alterations. However, more studies are warranted to dissect the mechanisms associated with these effects.

## Author Contributions

CdA, RL, LO, and VC-S: performed the experiments and wrote the paper; RG and CR: provided scientific advice and revised the paper; JM, AS, and RO: designed the scientific concept, provided financial support, wrote and revised the paper.

### Conflict of Interest Statement

The authors declare that the research was conducted in the absence of any commercial or financial relationships that could be construed as a potential conflict of interest.

## Abbreviations

WHO: World Health Organization
BBB: Blood-brain barrier
RBD: Regional Basic Diet
HFD: High Fat Diet
PFA: Paraformaldehyde
PBS: Phosphate buffered saline
PVDF: Polyvinylidene difluoride membrane
DEEPPD: Diethyl-pera-phenylenediamine
H_2_O_2_: Hydrogen Peroxide
TBAR: Thiobarbituric Acid Reactive
MDA: Malondialdehyde
PND: Pos-natal day
GFAP: Glial Fibrillary Acidic Protein
MPO: Myelloperoxidase.

## References

[1] Walson JL Berkley JA. The impact of malnutrition on childhood infections. Curr Opin Infect Dis. (2018) 31:231–6. 10.1097/QCO.0000000000000448 29570495 PMC6037284

[2] Ferreira HS Moura FA Cabral CR Jr Florencio TM Vieira RC de Assuncao ML. Short stature of mothers from an area endemic for undernutrition is associated with obesity, hypertension and stunted children: a population-based study in the semi-arid region of Alagoas, Northeast Brazil. Br J Nutr. (2009) 101:1239–45. 10.1017/S0007114508059357 19017417

[3] Ofori-Asenso R Agyeman AA Laar A Boateng D. Overweight and obesity epidemic in Ghana-a systematic review and meta-analysis. BMC Public Health (2016) 16:1239. 10.1186/s12889-016-3901-4 27938360 PMC5148846

[4] Pulgaron ER. Childhood obesity: a review of increased risk for physical and psychological comorbidities. Clin Ther. (2013) 35:A18–32. 10.1016/j.clinthera.2012.12.014 23328273 PMC3645868

[5] Tshala-Katumbay D Mwanza JC Rohlman DS Maestre G Oria RB. A global perspective on the influence of environmental exposures on the nervous system. Nature (2015) 527:S187–92. 10.1038/nature16034 26580326 PMC4772865

[6] Katz DL. The mass of humanity and the weight of the world: obesity and the environment at a confluence of causes. Curr Obes Rep. (2016) 5:386–8. 10.1007/s13679-016-0236-5 27739006

[7] Cardoso FL Brites D Brito MA. Looking at the blood-brain barrier: molecular anatomy and possible investigation approaches. Brain Res Rev. (2010) 64:328–63. 10.1016/j.brainresrev.2010.05.003 20685221

[8] Lotocki G de Rivero Vaccari JP Perez ER Sanchez-Molano J Furones-Alonso O Bramlett HM. Alterations in blood-brain barrier permeability to large and small molecules and leukocyte accumulation after traumatic brain injury: effects of post-traumatic hypothermia. J Neurotrauma (2009) 26:1123–34. 10.1089/neu.2008.0802 19558276 PMC2848945

[9] Hersoug LG Moller P Loft S. Role of microbiota-derived lipopolysaccharide in adipose tissue inflammation, adipocyte size and pyroptosis during obesity. Nutr Res Rev. (2018) 31:153–63. 10.1017/S0954422417000269 29362018

[10] Ling PR Smith RJ Kie S Boyce P Bistrian BR. Effects of protein malnutrition on IL-6-mediated signaling in the liver and the systemic acute-phase response in rats. Am J Physiol Regul Integr Comp Physiol. (2004) 287:R801–8. 10.1152/ajpregu.00715.2003 15371280

[11] DeBoer MD Lima AA Oria RB Scharf RJ Moore SR Luna MA. Early childhood growth failure and the developmental origins of adult disease: do enteric infections and malnutrition increase risk for the metabolic syndrome? Nutr Rev. (2012) 70:642–53. 10.1111/j.1753-4887.2012.00543.x 23110643 PMC3493112

[12] Oria RB Murray-Kolb LE Scharf RJ Pendergast LL Lang DR Kolling GL. Early-life enteric infections: relation between chronic systemic inflammation and poor cognition in children. Nutr Rev. (2016) 74:374–86. 10.1093/nutrit/nuw008 27142301 PMC4892302

[13] Honda H Ueda M Kojima S Mashiba S Suzuki H Hosaka N. Oxidized high-density lipoprotein is associated with protein-energy wasting in maintenance hemodialysis patients. Clin J Am Soc Nephrol. (2010) 5:1021–8. 10.2215/CJN.06110809 20395357 PMC2879312

[14] Chen TG Chen TL Chang HC Tai YT Cherng YG Chang YT. Oxidized low-density lipoprotein induces apoptotic insults to mouse cerebral endothelial cells via a Bax-mitochondria-caspase protease pathway. Toxicol Appl Pharmacol. (2007) 219:42–53. 10.1016/j.taap.2006.11.031 17239413

[15] Ueno PM Oria RB Maier EA Guedes M de Azevedo OG Wu D. Alanyl-glutamine promotes intestinal epithelial cell homeostasis *in vitro* and in a murine model of weanling undernutrition. Am J Physiol Gastrointest Liver Physiol. (2011) 301:G612–22. 10.1152/ajpgi.00531.2010 21799183 PMC3191556

[16] Almeida-Suhett CP Scott JM Graham A Chen Y Deuster PA. Control diet in a high-fat diet study in mice: regular chow and purified low-fat diet have similar effects on phenotypic, metabolic, and behavioral outcomes. Nutr Neurosci. (2017) 22:19–28. 10.1080/1028415X.2017.1349359 28721750

[17] Coelho-Santos V Leitao RA Cardoso FL Palmela I Rito M Barbosa M. The TNF-alpha/NF-kappaB signaling pathway has a key role in methamphetamine-induced blood-brain barrier dysfunction. J Cereb Blood Flow Metab. (2015) 35:1260–71. 10.1038/jcbfm.2015.59 25899299 PMC4528012

[18] Leitao RA Sereno J Castelhano JM Goncalves SI Coelho-Santos V Fontes-Ribeiro C. Aquaporin-4 as a new target against methamphetamine-induced brain alterations: focus on the neurogliovascular unit and motivational behavior. Mol Neurobiol. (2018) 55:2056–69. 10.1007/s12035-017-0439-0 28283882

[19] Tavares G Martins M Correia JS Sardinha VM Guerra-Gomes S das Neves SP. Employing an open-source tool to assess astrocyte tridimensional structure. Brain Struct Funct. (2017) 222:1989–99. 10.1007/s00429-016-1316-8 27696155 PMC5406431

[20] Liebner S Dijkhuizen RM Reiss Y Plate KH Agalliu D Constantin G. Functional morphology of the blood-brain barrier in health and disease. Acta Neuropathol. (2018) 135:311–36. 10.1007/s00401-018-1815-1 29411111 PMC6781630

[21] Nag S Manias JL Stewart DJ. Expression of endothelial phosphorylated caveolin-1 is increased in brain injury. Neuropathol Appl Neurobiol. (2008) 35:417–26. 10.1111/j.1365-2990.2008.01009.x 19508446

[22] Kuo YC Lu CH. Effect of human astrocytes on the characteristics of human brain-microvascular endothelial cells in the blood-brain barrier. Colloids Surf B Biointerfaces (2011) 86:225–31. 10.1016/j.colsurfb.2011.04.005 21524890

[23] Cekanaviciute E Buckwalter MS. Astrocytes: integrative regulators of neuroinflammation in stroke and other neurological diseases. Neurotherapeutics (2016) 13:685–701. 10.1007/s13311-016-0477-8 27677607 PMC5081110

[24] Kearney J. Food consumption trends and drivers. Philos Trans R Soc Lond B Biol Sci. (2010) 365:2793–807. 10.1098/rstb.2010.0149 20713385 PMC2935122

[25] Benardais K Gudi V Gai L Nessler J Singh V Prajeeth CK. Long-term impact of neonatal inflammation on demyelination and remyelination in the central nervous system. Glia (2014) 62:1659–70. 10.1002/glia.22706 24909143

[26] Hsu TM Kanoski SE. Blood-brain barrier disruption: mechanistic links between Western diet consumption and dementia. Front Aging Neurosci. (2014) 6:88. 10.3389/fnagi.2014.00088 24847262 PMC4023063

[27] Blanton LV Barratt MJ Charbonneau MR Ahmed T Gordon JI. Childhood undernutrition, the gut microbiota, and microbiota-directed therapeutics. Science (2016) 352:1533. 10.1126/science.aad9359 27339978

[28] Schachter J Martel J Lin CS Chang CJ Wu TR Lu CC. Effects of obesity on depression: a role for inflammation and the gut microbiota. Brain Behav Immun. (2018) 69:1–8. 10.1016/j.bbi.2017.08.026 28888668

[29] Spencer SJ D'Angelo H Soch A Watkins LR Maier SF Barrientos RM. High-fat diet and aging interact to produce neuroinflammation and impair hippocampal- and amygdalar-dependent memory. Neurobiol Aging (2017) 58:88–101. 10.1016/j.neurobiolaging.2017.06.014 28719855 PMC5581696

[30] Stangl D Thuret S. Impact of diet on adult hippocampal neurogenesis. Genes Nutr. (2009) 4:271–82. 10.1007/s12263-009-0134-5 19685256 PMC2775886

[31] Alaverdashvili M Caine S Li X Hackett MJ Bradley MP Nichol H. Protein-energy malnutrition exacerbates stroke-induced forelimb abnormalities and dampens neuroinflammation. Transl Stroke Res. (2018) 9:622–30. 10.1007/s12975-018-0613-3 29397529

[32] Smith SE Figley SA Schreyer DJ Paterson PG. Protein-energy malnutrition developing after global brain ischemia induces an atypical acute-phase response and hinders expression of GAP-43. PLoS ONE (2014) 9:e107570. 10.1371/journal.pone.0107570 25259609 PMC4178032

[33] de Queiroz CA Fonseca SG Frota PB Figueiredo IL Aragao KS Magalhaes CE. Zinc treatment ameliorates diarrhea and intestinal inflammation in undernourished rats. BMC Gastroenterol. (2014) 14:136. 10.1186/1471-230X-14-136 25095704 PMC4142448

[34] Sellmann C Priebs J Landmann M Degen C Engstler AJ Jin CJ. Diets rich in fructose, fat or fructose and fat alter intestinal barrier function and lead to the development of nonalcoholic fatty liver disease over time. J Nutr Biochem. (2015) 26:1183–92. 10.1016/j.jnutbio.2015.05.011 26168700

[35] Zhou T Zhao L Zhan R He Q Tong Y Tian X. Blood-brain barrier dysfunction in mice induced by lipopolysaccharide is attenuated by dapsone. Biochem Biophys Res Commun. (2014) 453:419–24. 10.1016/j.bbrc.2014.09.093 25268765

[36] Ullen A Singewald E Konya V Fauler G Reicher H Nusshold C. Myeloperoxidase-derived oxidants induce blood-brain barrier dysfunction *in vitro* and *in vivo*. PLoS ONE (2013) 8:e64034. 10.1371/journal.pone.0064034 23691142 PMC3653856

[37] Singh AK Jiang Y How does peripheral lipopolysaccharide induce gene expression in the brain of rats? Toxicology (2004) 201:197–207. 10.1016/j.tox.2004.04.015 15297033

[38] Yang CH Kao MC Shih PC Li KY Tsai PS Huang CJ. Simvastatin attenuates sepsis-induced blood-brain barrier integrity loss. J Surg Res. (2015) 194:591–8. 10.1016/j.jss.2014.11.030 25534234

[39] Kanoski SE Zhang Y Zheng W Davidson TL. The effects of a high-energy diet on hippocampal function and blood-brain barrier integrity in the rat. J Alzheimers Dis. (2010) 21:207–19. 10.3233/JAD-2010-091414 20413889 PMC4975946

[40] Tucsek Z Toth P Sosnowska D Gautam T Mitschelen M Koller A. Obesity in aging exacerbates blood-brain barrier disruption, neuroinflammation, and oxidative stress in the mouse hippocampus: effects on expression of genes involved in beta-amyloid generation and Alzheimer's disease. J Gerontol A Biol Sci Med Sci. (2014) 69:1212–26. 10.1093/gerona/glt177 24269929 PMC4172034

[41] Wanrooy BJ Kumar KP Wen SW Qin CX Ritchie RH Wong CHY. Distinct contributions of hyperglycemia and high-fat feeding in metabolic syndrome-induced neuroinflammation. J Neuroinflammation (2018) 15:293. 10.1186/s12974-018-1329-8 30348168 PMC6198529

[42] Denver P Gault VA McClean PL. Sustained high-fat diet modulates inflammation, insulin signalling and cognition in mice and a modified xenin peptide ameliorates neuropathology in a chronic high-fat model. Diabetes Obes Metab. (2018) 20:1166–75. 10.1111/dom.13210 29316242

[43] Feoli AM Leite MC Tramontina AC Tramontina F Posser T Rodrigues L. Developmental changes in content of glial marker proteins in rats exposed to protein malnutrition. Brain Res. (2008) 1187:33–41. 10.1016/j.brainres.2007.10.035 18021757

[44] Liddelow SA Barres BA. Reactive astrocytes: production, function, and therapeutic potential. Immunity (2017) 46:957–67. 10.1016/j.immuni.2017.06.006 28636962

[45] Liddelow SA Guttenplan KA Clarke LE Bennett FC Bohlen CJ Schirmer L. Neurotoxic reactive astrocytes are induced by activated microglia. Nature (2017) 541:481–7. 10.1038/nature21029 28099414 PMC5404890

[46] Zamanian JL Xu L Foo LC Nouri N Zhou L Giffard RG. Genomic analysis of reactive astrogliosis. J Neurosci. (2012) 32:6391–410. 10.1523/JNEUROSCI.6221-11.2012 22553043 PMC3480225

